# A Novel ssDNA Aptamer Targeting Carcinoembryonic Antigen: Selection and Characterization

**DOI:** 10.3390/biology11101540

**Published:** 2022-10-20

**Authors:** Nigara Yunussova, Marzhan Sypabekova, Zhazira Zhumabekova, Bakhyt Matkarimov, Damira Kanayeva

**Affiliations:** 1 Ph.D. Program in Life Sciences, Nazarbayev University, 53 Kabanbay Batyr Ave., Astana 010000, Kazakhstan; 2 National Laboratory Astana, Nazarbayev University, 53 Kabanbay Batyr Ave., Astana 010000, Kazakhstan; 3 M.Sc. Program in Biological Sciences, Nazarbayev University, 53 Kabanbay Batyr Ave., Astana 010000, Kazakhstan; 4 Department of Biology, School of Sciences and Humanities, Nazarbayev University, 53 Kabanbay Batyr Ave., Astana 010000, Kazakhstan

**Keywords:** aptamer, carcinoembryonic antigen (CEA), selection, SELEX, ELONA, bioinformatics analysis, HT-29 human colon adenocarcinoma

## Abstract

**Simple Summary:**

Carcinoembryonic antigen (CEA), a well-known cancer biomarker that is used to diagnose various cancers, notably colorectal cancer, was used as a target to select and characterize single-stranded DNA aptamers. These short and compact nucleic acid sequences, known as aptamers, are becoming ever more popular in both therapeutics and diagnostics. In this study, SELEX and NGS techniques were employed in the aptamer selection processes. Aptamer characterization techniques, such as enzyme-linked oligonucleotide assay (ELONA), dot blot, and bioinformatics assays, were conducted to observe the affinity binding of the selected aptamer to the target protein. Confocal microscopy results demonstrated how the fluorescently labelled aptamer sequence recognized CEA, which is expressed in HT-29 human colon adenocarcinoma cell line. These findings show that the selected aptamer has the potential to be employed as a recognition component in rapid detection systems, such as aptasensors.

**Abstract:**

One of the major causes of a drastically shorter life expectancy and one of the most prevalent diseases in the world today is cancer. Given the data on the rise in cancer cases throughout the world, it is obvious that, despite the diagnostic techniques currently being used, there is a pressing need to develop precise and sensitive techniques for early diagnosis of the disease. A high degree of affinity and specificity towards particular targets is maintained by the short nucleic acid molecules known as aptamers. Aptamers outperform antibodies due to their unique benefits, such as their simplicity in synthesis and modification, lack of toxicity, and long-term stability. Utilizing an accurate recognition element and a robust signal transduction mechanism, molecular diagnostics can be extremely sensitive and specific. In this study, development of new single-stranded DNA aptamers against CEA for use in cancer diagnostics was accomplished using SELEX and NGS methods. As a result of 12 iterative SELEX rounds, nine aptamer candidates against CEA were developed. NGS comparative analysis revealed that round twelve had an enriched number of aptamers that were specifically bound, as opposed to round eight. Among the selected nine sequences characterized by bioinformatics analysis and ELONA, an aptamer sequence with the highest specificity and affinity for the target protein was identified and further examined. Aptamer sequence (6) was screened in a concentration-dependent assay, specificity analysis was performed, and its potential secondary and tertiary structures were predicted, which enabled us to test one of the possible putative interactions with CEA. Finally, aptamer sequence (6) labelled with a Cy5 fluorescent tag was used in confocal microscopy to observe its binding towards the CEA expressed in HT-29 human colon adenocarcinoma cell line.

## 1. Introduction

Cancer is a genetic disease that involves uncontrolled growth of cells with the ability to invade other parts of the body [1]. According to the World Health Organization (WHO), cancer is the second leading cause of death worldwide, and it was estimated that every sixth cause of death is due to cancer, with lung, liver, colon, and breast cancers making up the majority of deaths [2]. Successful cancer treatment heavily depends on early cancer detection through screening or by laboratory blood or urine tests [3,4]. However, the current diagnostic techniques have limitations, such as high cost, scarcity of providers, inaccessibility of screening in rural areas [5], and inaccuracy of the tests [6]. Thus, to combat these issues, new effective diagnostic methods must be developed on a continuous basis. Biomarkers have had enormous significance in cancer screening, diagnosis, and therapy [7]. One of the most prominent cancer biomarkers is carcinoembryonic antigen (CEA)**,** which is highly expressed in colon, gastric, breast [8], and lung cancer [9]. The normal range of serum CEA in healthy people is 0–5 ng/mL [10]. CEA contributes to carcinogenesis by enhancing cancer cell adhesion, disrupting cell polarity, and triggering anoikis [11], a form of programmed cell death. CEA level can be employed in cancer diagnosis, recovery prognosis, cancer recurrence, metastasis, and assessing the outcome of chemotherapy treatment. For example, a study conducted by the authors of Ref. [12] involved a meta-analysis of diagnostic value and prognostic significance of CEA to anticipate poor prognosis in clinical diagnosis of pancreatic cancer patients. The cumulative specificity and sensitivity values for serum CEA alone were 0.82 (95% confidence interval (CI), 0.79–0.84), and 0.43 (95% CI, 0.39–0.47), respectively. In terms of prognostic significance, CEA expression was highly correlated with overall survival in patients with pancreatic cancer [12]. Research conducted in Ref. [13] reported that increased levels of tumor burden indicators CEA and carbohydrate antigen 19-9 (CA19-9) are similarly linked with poorer prognosis. Hence, according to Ref. [14], a linear combination of CEA and CA19-9 is much more accurate in predicting prognosis than either tumor marker alone. There are enough established methods for CEA detection at the moment, such as enzyme immunoassay (limit of detection (LOD) 0.5 ng/mL) [15] and fluoroimmunoassay (LOD 1.0 ng/mL) [16]. However, these assays employ antibodies that have significant disadvantages, including high immunogenicity, high cost [17], and short-term stability [18]. Therefore, immunoassays should be phased out in favor of more efficient alternatives.

Aptamers are the most promising potential antibody substitute. These chemically synthesized oligonucleotides are single-stranded DNA or RNA featuring precise specificity and affinity for a variety of targets by reason of their three-dimensional folding and target recognition [19,20,21]. Even though antibodies are well known to the world, aptamers are a superior alternative for a number of applications due to their particular benefits, where ease of different chemical modifications, high consistency, and cost-effectiveness are the most notable ones [22,23]. Oligonucleotides are synthesized in a laboratory without use of complicated cell or animal studies [24] from a large pool of nucleic acid sequences through an in vitro selection process known as systematic evolution of ligands by exponential enrichment (SELEX) [25]. According to research [20,26], a target of interest is incubated with a pool of random aptamers, and, following amplification, aptamers with low affinity to the target are eliminated from the pool, leaving only possible aptamer candidates with high affinity [27]. 

Next generation sequencing (NGS) is used to further reveal aptamer sequences that have demonstrated heavy use in the past ten years [28,29]. The sequencing consists of four main steps: template preparation, sequencing, imaging, and data analysis [30]. The principle behind NGS is that, first, the DNA is randomly cut into smaller sizes, and the produced template is immobilized onto a solid surface. Then, the templates are amplified, followed by sequencing reactions and imaging [29]. However, other sequencing methods can be employed, such as in Ref. [31], where researchers cloned PCR products into *Escherichia coli* via electroporation for sequencing. Detection and evaluation of the target protein can be performed by techniques such as enzyme-linked oligonucleotide assay (ELONA) and surface plasmon resonance (SPR), as in Refs. [32,33]. ELONA is a modification of enzyme-linked immunosorbent assay (ELISA), in which an antibody is replaced with an aptamer [32,33,34,35], where the binding interaction between a target and an aptamer sequence can be investigated [33]. 

Bioinformatics analysis aids in the study of ssDNA aptamers’ secondary and tertiary structures, which is critical in understanding aptamer–target interactions [36]. Once the tertiary structures of aptamers are analyzed, the next step is to apply molecular docking, which is a computational approach that uses the lowest Δ*G* binding energy to predict development of an aptamer–target complex [37,38]. The structural features of the target molecules and aptamers determine how effective the complex interaction is [39]. Aptamers can take on a number of structural patterns [40] and can attach to their targets by hydrogen bonding, hydrophobic interactions, electrostatic interactions, protein stacking, van der Waals forces, or a combination of these forces [41]. Another study [42] presents results on tertiary structure prediction of aptamers targeting prostate cancer cells and molecular docking simulations with cathepsin B (CatB) molecule. Confocal microscopy analysis is widely used because of its simplicity and flexibility [43]. According to Ref. [44], only in the case of high affinity binding between aptamers and the marker can a high-resolution image be obtained. Typically, this type of imaging is achieved with fluorescently labelled aptamers that specifically bind to a biomarker of interest, whereby binding events to a protein can be observed through a fluorescent dye [44].

In this research, ssDNA aptamers against CEA were selected as a result of twelve rounds of SELEX technique, where NGS comparative analysis showed that, at round twelve, the number of specifically bound aptamers was enriched compared to round eight. Occurrence analysis of short motifs showed, overall, eight-hundred-ninety-six sequence families, of which the most frequent nine families were chosen. Nine potential CEA aptamer candidates were evaluated and characterized using a dot blot assay and ELONA. A bioinformatics approach represented aptamer secondary and tertiary structures and aptamer–CEA complex molecular docking analysis. Confocal microscopy analysis was used to visualize the binding between fluorescently labelled aptamer (6) and the target CEA.

## 2. Materials and Methods

### 2.1. Proteins 

CEA from human fluids (≥95% SDS-PAGE) was obtained from Sigma-Aldrich (St. Louis, MO, USA) as a buffered aqueous solution containing 0.1% sodium azide. Prior to SELEX, the antigen was dialyzed in PBS (0.01 M phosphate buffer, 0.027 M potassium chloride, 0.137 M sodium chloride, pH 7.5). This was completed to remove sodium azide that could potentially alter binding capabilities of ssDNA aptamers to CEA. Non-target proteins: (i) IL-6 human recombinant was obtained from Merck (Darmstadt, Germany) as a lyophilized powder; (ii) purified from normal human serum, native human serum albumin (HSA) (>95% SDS-PAGE) was obtained from Abcam (Cambridge, UK) as a liquid. Biotin (≥ 99% HPLC) was obtained from Sigma-Aldrich (St. Louis, MO, USA) as a lyophilized powder. Bovine serum albumin (BSA, BPE1600-100) was purchased from Fisher Scientific (Waltham, MA, USA, USA). 

### 2.2. DNA Library, Primers

The oligonucleotide template was synthesized as a single-stranded 80-mer with the following sequence: 5′-ACG ACG AGA TGG ACT GTA AG—N40—CTC CAA TGC TAG TCG CAA GT-3′, where the central 40 nucleotides represent random oligonucleotides based on equal incorporation of A, T, G, and C at each position. The dsDNAs were obtained by PCR amplification using forward 5′-TCA CTT CAA ATG TGC GCT TC-3′ and reverse 5′-TTC TAC CCC CTG TTT TGA CG-3′ primers. The library and primers were synthesized, and PAGE purified by National Biotechnology Center (Astana, Kazakhstan), whereas biotinylated and phosphorylated primers were synthesized by Sigma-Aldrich (Germany).

### 2.3. Cell Lines 

HT-29 human colon adenocarcinoma (ATCC HTB-38) cell line and HeLa S3 (ATCC CCL-2.2) cell line were obtained from American Type Culture Collection (ATCC, Manassas, VA, USA). Both cell lines were cultured in Dulbecco’s modified eagle medium (DMEM, Gibco, NY, USA) supplemented with 10% fetal bovine serum (FBS, Millipore, Burlington, MA, USA) and antibiotic antimycotic solution (1% Pen/Strep, Sigma-Aldrich, USA) at 37 °C in a humidified incubator (Binder KT53, Tuttlingen, Germany) containing 5% CO_2_. All manipulations with cell cultures were carried out in a Class II Biological Safety Cabinet (51026946, S2020 0.9 type, Thermo-Scientific, Waltham, MA, USA). 

### 2.4. In Vitro Selection of Aptamers

**Figure 1:**
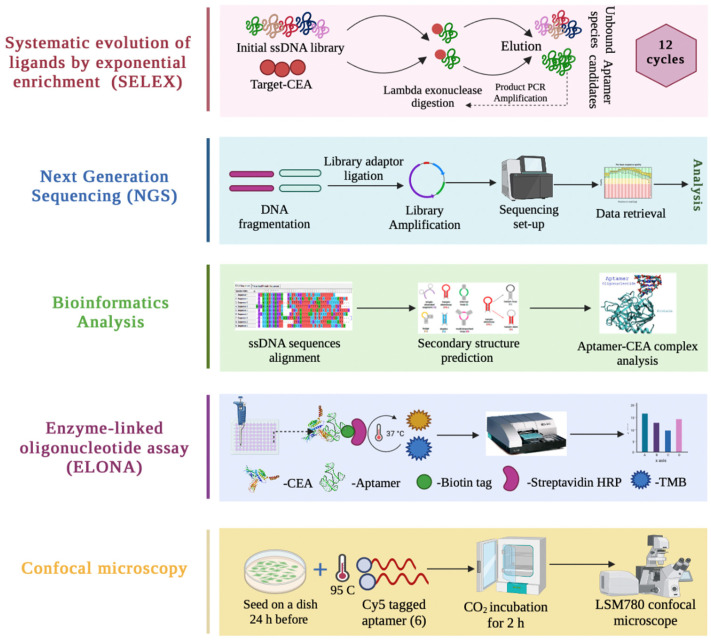
Schematic overview of CEA aptamer selection and characterization using SELEX, NGS, bioinformatics analysis, ELONA, and confocal microscopy steps.

As shown in Figure 1, aptamer candidates against CEA were selected using the SELEX technique [20] with modifications. The detailed protocol for SELEX procedure was performed as follows. To exclude filter-binding ssDNA sequences from the pool, the DNAs were passed 2–3 times prior to the selection cycle through a pre-wetted nitrocellulose acetate membrane (0.45 μm HAWP filter, Millipore, MA, USA) in a filter holder (“pop-top”, a diameter of 13 mm, Millipore). To initiate in vitro selection, ssDNA library was denatured at 95 °C for 10 min in a thermocycler (Mastercycler Gradient, Eppendorf) and was allowed to cool down to approximately 38 °C inside the thermocycler. Denatured DNAs were then incubated with the target protein at 15 rpm on a variable speed rotor (Grant Bio PTR-30, Keison) for 1 h and 45 min at room temperature. This reaction mixture was then filtered over a HAWP filter and washed with a binding buffer (50 mM Tris-HCl; 25 mM NaCl; 5 mM MgCl_2_; 10 mM DTT; pH 7.5). Further, ssDNAs that were retained with the protein on the filter were eluted with an elution buffer (0.4 mM sodium acetate, EDTA disodium salt; urea; pH 5.5) at 70–80 °C for 5 min two times. Afterwards, the eluted DNA was diluted with an equal volume of deionized water (dH_2_O) and was precipitated (0.12 mg glycogen, equal volume of 7.5 M ammonium acetate, and 1 mL of 95% ethanol) and incubated for 1 h at −80 °C. After centrifugation at 13,000 rpm at 4 °C for 1 h, supernatant was discarded, and pellet was washed twice with 75% ethanol solution and resuspended in dH_2_O for further PCR. Amplification conditions were as follows: initial denaturation at 95 °C for 5 min, 15 cycles of denaturation at 92 °C for 15 s, annealing at 50 °C for 30 s, and extension at 72 °C for 15 s. The number of PCR cycles was decreased from 15 to 12 after SELEX cycle 8. Amplified products were analyzed on 11% acrylamide-bis (Bio-Rad) gel with SeperateIT (Applichem) solutions at 150 V for 20–30 min after binding with SYBR Green 1 (Invitrogen, Waltham, MA, USA). Further, ssDNA was obtained by digestion of PCR products amplified with phosphorylated reverse primers with 5 units of lambda exonuclease (New England Biolabs, Ipswich, MA, USA) per 50 µL reaction. Digested products were precipitated as above (resuspension of pellet in binding buffer not in water) and used for the next round of SELEX. A total of 12 iterations of SELEX were performed. For additional rounds of selection, the amount of protein was reduced each cycle in the binding buffer (total volume of 25 µL). Incubation time was in a range from 1 h 45 min for the first cycle and 10 min for the last. Incubation conditions as well as the amount of target protein and ssDNA are listed in Table 1. The molar ratio of CEA and ssDNA was adjusted so that it decreased after each cycle starting from cycle 7. The concentration of ssDNA was measured using a NanoDrop 8000 Spectrophotometer (ThermoScientific, Wilmington, DE, USA). All procedures (where applicable) were performed in a biosafety level 2 cabinet (Purifier Delta Series Class II, Type A2 Biological Safety Cabinet, Thermo Scientific, Wilmington, DE, USA).

### 2.5. Next Generation Sequencing and Bioinformatics Analysis 

SELEX products were amplified using specially designed primers, which were sequenced using the NGS on an Illumina Hiseq Rapid Mode system in a single read mode (AptaIT, Munich, Germany). Briefly, two sets of NGS primers were designed and synthesized (Nazarbayev University, Kazakhstan) by adding indexes GAT CAG and TAG CTT to the 5′-end of SELEX primer sequence of forward- and reverse primer. SELEX products of respective rounds were then PCR amplified using the Taq Core Kit (Qiagen, Hilden, Germany), and 1 µL of ssDNA 12th round (conc. 93.1 ng/µL) and 1 µL of ssDNA 8th round (conc. 21 ng/µL) were used as a template for amplification. The PCR products were then purified using Mini Elute PCR purification Kit (Qiagen) and GE NAP-5 columns (GE Healthcare, Chicago, IL, USA). Concentration and purity measurements of each indexed probe were obtained by a Nanodrop 2000 spectrophotometer (Thermofisher, Waltham, MA, USA). FASTQ data obtained from the NGS study were then subjected to an in silico analysis that included parsing of raw data, basic statistics of naïve and enriched libraries, and identification of aptamer families in selection rounds. Occurrence and full-length sequences in selection rounds to assess in which rounds aptamers evolved and competed against each other were viewed using the COMPAS-viewer software. 

Multiple aptamer sequence alignment was performed using ClustalX2 software [45]. Based on the sequence alignment results, a phylogenetic tree of the studied aptamers was constructed using Dendroscope 3 software [46]. Secondary structures of aptamer sequences and the dot-bracket formats of aptamer sequences were predicted and obtained by the RNAstructureWeb online server (version 6.4, https://rna.urmc.rochester.edu/RNAstructureWeb/), which incorporates predicting a minimum free energy (MFE) structure, finding structures with maximum expected accuracy, and pseudoknot prediction. The obtained dot-bracket formats of aptamer sequences were further utilized as input for the RNAComposer web server [47] (v.1.0, https://rnacomposer.cs.put.poznan.pl) to build the three-dimensional structure of CEA aptamer sequences. The three-dimensional structures of the RNA forms were modified using Discovery Studio Visualizer software (v.21.1.0.20298) since the output of RNAComposer was RNA form of the ssDNA sequences. Uracil bases were changed to thymine bases and ribose units to deoxyribose units. The PDB format of CEA (ID: 2QSQ, https://www.rcsb.org/structure/2QSQ, accessed on 25 September 2021) [48] and ssDNA sequences, which were defined as the respective ligand and receptor, were used for molecular docking simulation. Molecular docking simulation was performed in ZDOCK (v.3.0.2, https://zdock.umassmed.edu, accessed on 25 September 2021) online platform. The visualization files were further observed using Discovery Studio Visualizer software. 

### 2.6. Affinity Analysis 

SELEX cycle pools were evaluated for their binding to the target CEA using ELONA based on protocol [49] with modifications (Figure 1). Biotin served as a positive control, and dH_2_O as a negative control. Each biotinylated ssDNA pool was generated using asymmetric PCR with prevalent concentrations of biotinylated forward primer (20 µM) and less concentrated reverse primer (0.8 µM). The PCR conditions were the same as used for SELEX. After PCR, the ssDNA biotinylated DNA pool was precipitated as described previously and concentration was measured on a Nanodrop 2000 spectrophotometer. Ninety-six-well plates (Bioster, A947978) were coated with 500 ng of protein in a 100 µL of 100 mM Na_2_CO_3_ buffer overnight at 4 °C. After blocking with Superblock solution in PBS (Fisher Scientific, BPE1600-100) for 1 h at 4 °C, the wells were washed 4 times with 1× TBS buffer (24.2 g Tris Base, 80 g NaCl, pH 7.6), and biotin-labeled ssDNA aptamer pools from the SELEX cycles of 12, 10, and 8 (500 nM) were added to each well and incubated for 2 h at 37 °C. The wells were then washed 4 times with 1× TBS. Streptavidin-horseradish peroxidase conjugate (Thermo Scientific, NE170004) was diluted in 1:1000 in 1× TBS, and 100 µL was applied and incubated for 30 min at 37 °C. Then, 50 µL of turbo-3,3′5,5′-tetramethylbenzene (TMB, T8665, Sigma-Aldrich, St. Louis, MO, USA) was added to each well and incubated for 15 min at 37 °C. The reaction was quenched by adding 50 µL of 1 M H_2_SO_4_, and a protein–aptamer pool complex was quantified by determining the absorbance at 450 nm using a MultiScan FC spectrophotometer (Thermo Scientific, Waltham, MA, USA). For each sample, the optical density (*OD*) at 450 nm of background (a well incubated with dH_2_O) was subtracted from the *OD*_450_ value of an experimental sample. All measurements were carried out in triplicate, and the mean value of replicates, standard errors, and standard deviations from the mean were used to report the results.

ELONA was also used in (i) screening of relative binding events of selected nine ssDNA aptamers against CEA, (ii) in the concentration-dependent analysis of the relative binding affinity of the most sensitive aptamer, aptamer sequence (6), to the target CEA, (iii) specificity study, and iv) in the comparative binding analysis of aptamer (6) to the target CEA biotinylated at 3′- and 5′-termini, where the protocol described above was used with minor modifications. Protocol changes were as follows: target protein concentration was 300 ng, 5% BSA was used as a blocking solution, washing was performed 3 times, and aptamers were heated to 95 °C for 5 min before immobilization, then cooled on ice for 10 min. Aptamer sequences were synthesized and purified (HPLC) by Eurogentec (Seraing, Liege, Belgium). According to the manufacturer’s guidelines, diluted in nuclease-free water (Sigma-Aldrich, St. Louis, MO, USA), aptamer aliquots were kept at −20 °C until further use. A well with CEA but with no aptamer served as the negative control. Nuclease-free water was used as a background. To determine the specificity of the selected aptamer (6) to CEA, non-target proteins IL-6 and HSA were used. The *OD* value was measured at 450 nm with a spectrophotometer (BioTek Instruments, Winuskey, VT, USA). The results were analyzed with the Gen5 2.0 data analysis software (BioTek Instruments, Winuskey, VT, USA). Experiments were conducted in three technical replicates. For each sample, the optical density at 450 nm (*OD*) of background was subtracted from the *OD*_450_ value of the experimental sample. 

### 2.7. Confocal Microscopy

Confocal microscopy analysis was also performed to further confirm that aptamer sequence (6) binds specifically on the surface of CEA-positive cells but not CEA-negative cells (Figure 1). This technique was adopted from Ref. [50] with slight modifications. CEA-positive HT-29 cell line and CEA-negative HeLa S3 cell line were harvested at 60% confluency by detaching from the trypsin-treated plate. Cells were seeded on a FluoroDish cell culture dish (World Precision Instruments, Sarasota, FL, USA) 24 h prior to treatment with the Cy5-labeled aptamer (6). The medium was removed and incubated for 15 min at 37 °C in a binding buffer. The binding buffer consisted of the washing buffer with addition of 100 μg tRNA (Sigma-Aldrich, USA) and 1% BSA. The buffer was removed and washed twice with a washing buffer. The washing buffer was 1 X Dulbecco’s phosphate buffered saline (DPBS, 1X, Gibco, UK) with addition of 0.05 M of glucose (1000 PanReac AppliChem, Germany), 5 mL of 1 M MgCl_2_ (Sigma-Aldrich, USA). Then, Cy5-labeled aptamer (6) at a concentration of 250 nM was diluted in the binding buffer, and the cells were incubated with aptamer (6) for 2 h at 37 °C. Aptamer sequence (6) labelled with Cy5 at the 5′-terminus was synthesized by Eurogentec (Seraing, Liege, Belgium). Before incubation, aptamer (6) solution was heated at 95 °C for 5 min and cooled on ice for 10 min. After incubation, the aptamer solution was removed, and the cells were washed 3 times. Cells were visualized using a LSM 780 confocal laser scanning microscope (Carl Zeiss, Oberkochen, Germany), laser line: 633 nm: 4%. Obtained confocal microscopy micrographs were analyzed using ImageJ software (v.1.53) (US National Institutes of Health, Bethesda, MD, USA). 

### 2.8. Statistical Analysis 

All statistical analyses were performed by GraphPad Prism 9.1.0 for Windows (GraphPad Software, San Diego, CA, USA). Differences of *OD*_4__50_ across aptamers were tested by two-tailed *t*-test, ANOVA, and Kruskal–Wallis test. A nonlinear curve fitting analysis for dissociation constant (*K*_d_) calculation was performed. *K*_d_ of aptamer (6) was evaluated by measuring the absorbance at 450 nm. Data were expressed as mean ± standard deviation. Values *p* < 0.05 were considered statistically significant. 

## 3. Results

### 3.1. Selection of DNA Aptamers

**Figure 2:**
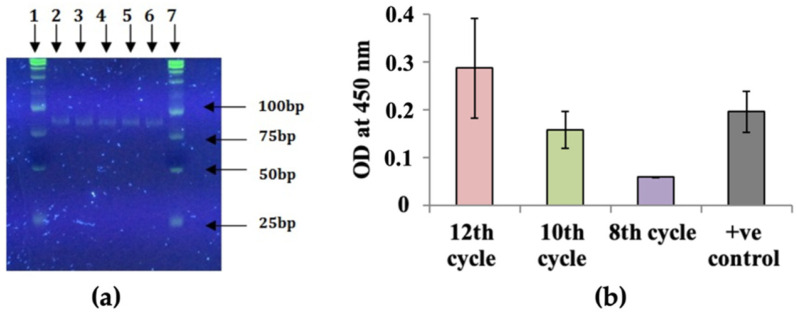
(**a**) 11% TBE-PAGE analysis of PCR products from cycle 9 of in vitro selection of aptamers against target CEA using SELEX stained with SYBR Green I: lanes 1 and 7: 25 bp DNA ladder; lanes 2–6 correspond to 5 different PCR tubes containing the same cycle 9 dsDNA product; (**b**) binding affinity of SELEX 12, 10, and 8 cycle pools to the target CEA. Binding of biotinylated ssDNA aptamer pools to the target CEA was determined by ELONA. Biotin served as “+” control. Each aptamer pool (500 nM) was incubated in wells coated with the target CEA. All data are shown as the means of ±SEMs (n = 3). The background was subtracted from the *OD*_450_ value of the experimental sample.

We performed in vitro selection with a pool of ssDNA sequences totally randomized at 40 nt to select DNA aptamers that bind to CEA. A commercially available human CEA (CEACAM5) was used as the target in this study. The ssDNA library had about 10^14^ different molecules. CEA-binding DNA aptamers were selected by incubating CEA with the DNA pool and subsequent nitrocellulose filtration. CEA-bound aptamers were eluted from the filters and then amplified by PCR. Individual single-stranded aptamer DNAs were derived from an aliquot of this dsDNA stock by λ exonuclease digestion. The ssDNA was used as the input for the next selection cycle. All DNAs were quantified using a spectrophotometer and further analyzed by PAGE gel. A representative PAGE gel is presented in Figure 2a with an expected 80 bp DNA size of the ninth cycle of selection of CEA aptamers.

### 3.2. Affinity Analysis of SELEX Cycle Aptamer Pools

ELONA was performed after SELEX rounds for evaluation of binding affinity to the target CEA. In this regard, target 500 ng/mL CEA was applied onto a 96-well plate and incubated overnight. The wells were blocked with a blocking buffer, and, after subsequent washing steps in between, the wells were incubated with a biotinylated aptamer pool from the eighth, tenth, and twelfth SELEX cycles. A similar concentration of biotin served as a positive control, and deionized water served as a background control. The background was subtracted from the *OD*_450_ value of experimental samples. The results of ELONA are presented in Figure 2b. With the increase in selection cycles, the selected aptamer pools showed stronger binding to the target with the increase in *OD*_450_ value from 0.05, 0.158 to 0.285 for the eighth, tenth, and twelfth cycles, respectively. The *OD*_450_ value for the positive control biotin was 0.195 and was slightly lower compared to the 12th cycle, indicating the importance of a cycle number increase in affinity aptamer pools towards the target. The ELONA results were visually confirmed by the dot blot assay as well, where the CEA protein was applied onto a nitrocellulose membrane. Biotin served as a positive control, and deionized water served as a negative control. Blocking, washing, and further detection steps were the same as for ELONA. The interaction between CEA and biotinylated aptamer pool was observed as violet-stained dots on the nitrocellulose membrane (Figure S1). The intensity of the dots increased with increasing cycle number, indicating a stronger interaction between the target and aptamer pool.

### 3.3. NGS and Bioinformatics Analysis

**Figure 3:**
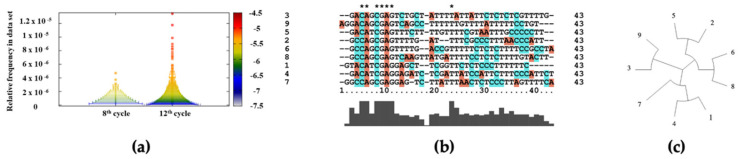
NGS and bioinformatics analysis of ssDNA aptamers. (**a**) Evolution of histograms of frequencies of data sequences over datasets with a bar-log (number of sequences with given frequency) and color-log (frequency) for SELEX rounds 8 and 12; (**b**) multiple sequences alignment of selected nine aptamer candidates; and (**c**) a circular cladogram of CEA aptamer candidates.

NGS probes were indexed via PCR and checked regarding quality control of samples, where the sizes (90 bp) of the amplified SELEX round eight and twelve products were confirmed on 3% agarose gel (Figure S2). The concentration and purity measurement of each indexed probe before NGS analysis is shown in Table S1. A comparison of the efficiency of CEA aptamer selection studies at high resolution is presented in Figure 3a, where the occurrence and frequency of individual clones from selection cycles eight and twelve were assessed. The color of the data point visualizes relative frequency according to the color ramp. The width of the data bars correlates with the number of clones at the given frequency. According to the histogram presented in Figure 3a, the frequency of rare sequences remains relatively the same from round eight to round twelve. According to the SELEX procedure, the number of rare sequences should decrease with the number of cycles. However, in the enriched round twelve, there were more unique clones (longer data bar, blue), indicating that, even after the twelfth cycle, there were still rare sequences in the DNA library present. In the direction of higher frequency (color changes from blue to red), the bars become narrower, representing that the number of clones that were present with a higher copy number decreased. The number of more frequent sequences appeared at round twelve compared to round eight, which means that the number of aptamers that specifically bound to CEA increased. Based on the presence of all consensus sequences identified in a co-occurrence mode, there were, overall, 896 sequence families, of which the nine most frequent families were identified. We then selected one of the most predominant DNA aptamers from each family for further analysis. 

Multiple aptamer sequence alignment was performed using Clustal2X software for the N40 sequences of nine aptamer candidates, as presented in Figure 3b. The aptamer candidates shared a considerable number of conserved sequences; particularly, seven alignment positions had identical nucleotides, which is more than 17% of the length of the largest aptamer sequence. Aptamers had the greatest similarity in the first half of the sequence from the 5’ terminus. A result of this observation is an indication of the non-random nature of the selection of these sequences in experimental procedures. Moreover, regions of maximum aptamer similarity were the most likely candidates for functional sites of protein–aptamer interaction. Based on the sequence alignment results, a phylogenetic tree of the studied aptamers was constructed using Dendroscope 3 software. Figure 3c demonstrates a circular cladogram of sequence similarity of N40 regions of CEA aptamer candidates. The cladogram indicates the similarity score of the aptamer sequences and shows how aptamers are related to each other. The presented circular cladogram is a complement to aptamer sequences’ multiple alignment (Figure 3b) by visualization of the aptamer’s (phylogenetic) tree based on alignment distance metrics [45,46].

### 3.4. Characterization of CEA Aptamers Using ELONA 

**Figure 4:**
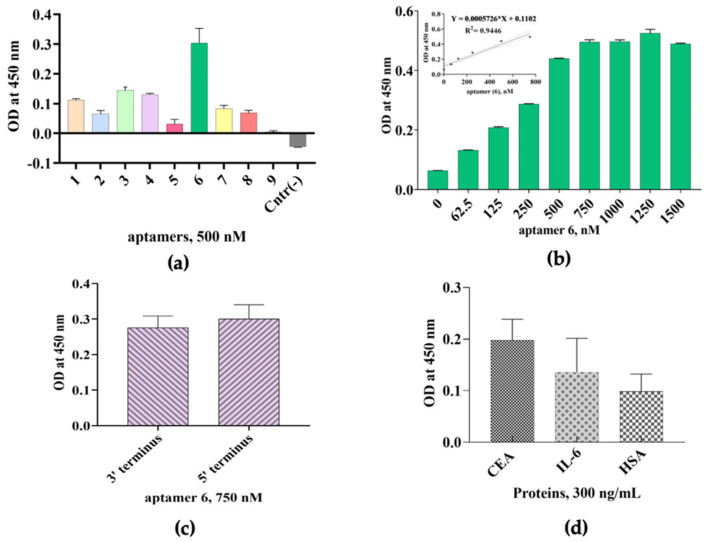
Binding capability and specificity study of the ssDNA aptamers towards CEA using ELONA. (**a**) Screening of the relative binding events of biotinylated aptamers against the target CEA. Nuclease free water was used as a background; (**b**) concentration-dependent analysis of the relative binding affinity of the biotinylated aptamer sequence (6) to the target CEA. Nuclease-free water was used as a background; (**c**) comparative binding analysis of aptamer (6) to the target CEA biotinylated at 3′-terminus and 5′- terminus. Nuclease-free water was used as a background; (**d**) specificity analysis of aptamer sequence (6). All *OD*_450_ values were subtracted from the background. All data are shown as the means of ± SEMs (n = 3).

We further screened nine aptamer sequences that were developed using the SELEX technology against the target CEA using ELONA. The binding affinity of aptamer candidates towards the target protein is shown in Figure 4a. A 96-well plate was coated with CEA overnight using a coating buffer, sodium carbonate, followed by an immobilization of aptamer sequences in a 500 nM concentration. Nuclease-free water was used as a background. Among the nine tested sequences, aptamer sequence (6) showed, significantly, the highest binding affinity towards CEA, with an *OD*_450_ of 0.303 (*p* = 0.01; ANOVA), while aptamer sequence (9) showed the lowest binding capability towards the CEA target protein (*OD*_450_ = 0.005) (Figure 4a). Based on the data obtained, aptamer (6) was chosen as the most sensitive aptamer towards CEA for further studies. The experiment was conducted in three technical replicates. For each sample, the *OD*_450_ of the background was subtracted from the experimental sample *OD*_450_ value. 

Screening of the relative binding events of the most sensitive CEA aptamer sequence, aptamer (6), is shown on Figure 4b, where the results of the concentration-dependent analysis of aptamer sequence (6) (0–1500 nM) against the target CEA are demonstrated. A well with nuclease-free water was used as a background. A concentration-dependent increase was found in the absorbance (*OD*_450_) for aptamer (6) in a concentration range between 0 nM and 1500 nM (*p* = 0.007; two-tailed *t*-test), with the surface saturation at 750 nM concentration (*OD*_450_ = 0.496). A linear correlation between *OD*_450_ value and aptamer sequence (6) in a concentration range of 0–750 nM was observed (Figure 4b inset), with the regression equation for *OD*_450_ change and aptamer sequence (6) concentration being *y* = 0.0005726*x* + 0.1102 with *R^2^* = 0.94, where *x* is the aptamer sequence (6) concentration in nM. *K*_d_ and maximum binding capacity (*B*_max_) of aptamer (6) was evaluated by measuring the absorbance at 450 nm. A nonlinear curve fitting analysis was conducted for the *K*_d_ calculation according to equation *Y* = *B*_max_ × *X*/ (*K*_d_ + *X*), where *B*_max_ was the maximal binding and *K*_d_ was the concentration of ligand required to reach half-maximal binding. Aptamer (6) showed a *K*_d_ value in the nanomolar range that equaled to 312.6 nM and *B*_max_ = 0.701. The experiment was conducted in three technical replicates.

A comparative binding analysis of aptamer (6) (750 nM) to the target CEA labelled with biotin at the 3′- and 5′-termini was also conducted (Figure 4c). Nuclease-free water was used as the background. It was found that the position of attachment of the biotin modification to different ends of the aptamer does not significantly (*p* = 0.5702; *t*-test) affect the binding efficiency and affinity of the aptamer to the protein (*OD*_450_ for 5′-aptamer (6) -[BtnTg]-3′ = 0.276 and *OD*_450_ for 5′-[BtnTg]-aptamer (6)-3′ = 0.3). 

Next, a specificity study was conducted, employing ELONA as well. CEA was used as the target, and two non-target proteins, IL-6 and HSA, were used in this assay (Figure 4d). All the proteins were tested in a concentration of 300 ng/mL. A well with nuclease-free water was used as a background. The data showed that, in the assayed conditions, aptamer sequence (6) recognized CEA target protein more (*p* = 0.0006; ANOVA) than the rest of the proteins, IL-6 and HSA. The signal output for CEA was *OD*_450_ = 0.198, for IL-6 *OD*_450_ = 0.129, and for HSA *OD*_450_ = 0.098. All the *OD* values were subtracted from the background. The experiment was conducted in three technical replicates.

**Figure 5:**
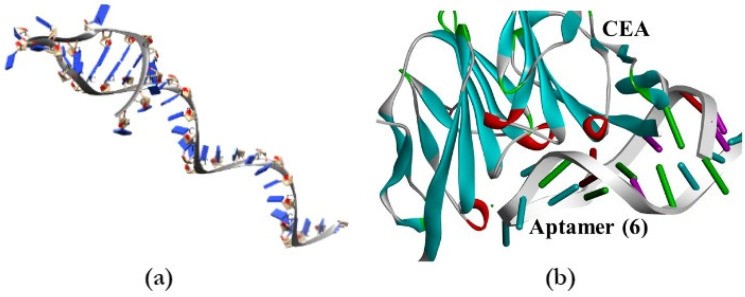
(**a**) Predicted tertiary structure of the aptamer sequence (6); (**b**) molecular docking simulation of an aptamer sequence (6) and CEA complex.

Bioinformatics analysis of aptamer sequence (6) was conducted, where secondary and tertiary structures were predicted and then applied for the molecular docking simulation analysis with the target CEA. We used predicted secondary structure of CEA aptamer sequence (6) to obtain a dot-bracket Vienna format from the RNAStructure online server to further generate a tertiary structure (Figure 5a) using the RNA Composer web server [51]. As the tertiary structure of an ssDNA sequence was obtained in a RNA form, it was necessary to modify it to DNA by changing the nucleotides and sugar rings using Discovery Studio Visualizer software. Further predicted aptamer sequence (6) structure was investigated on a putative binding with the target using the molecular docking simulation software. Figure 5b illustrates a simulation performed on the ZDOCK platform, where analysis of shape complementarity, electrostatic interactions, and statistical potential terms scoring were implemented to predict an aptamer–protein complex [52]. The obtained data were further visualized using Discovery Studio Visualizer software. Figure 5b demonstrates one of the possible putative interactions between aptamer (6) and the target CEA suggested by the docking platform. From the resulting complex of CEA–aptamer (6) (Figure 5b), we suppose that the protein is attached to a loop in the aptamer (6) structure, which corresponds to the conserved region in the sequence shown in the multiple alignment (Figure 3b). 

### 3.5. Confocal Microscopy 

**Figure 6:**
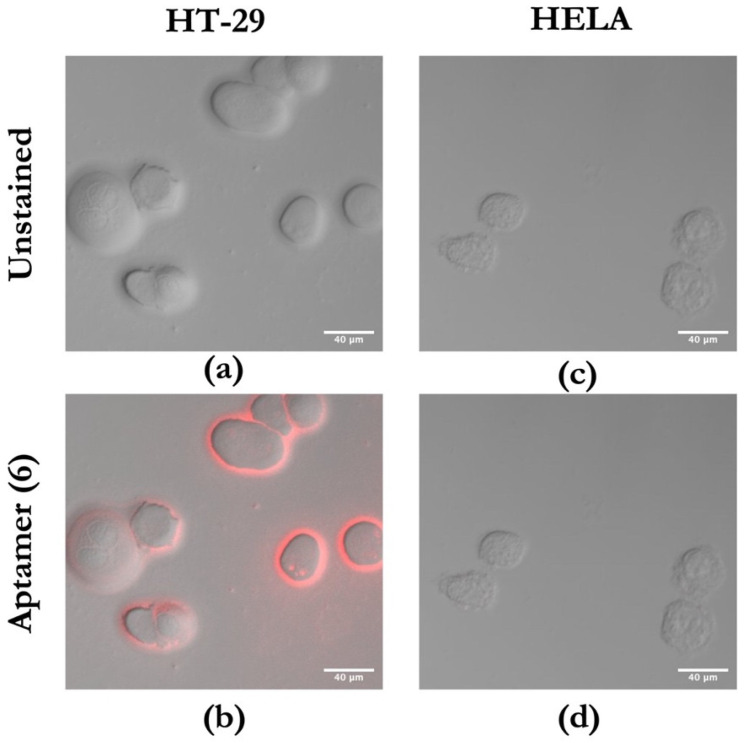
Confocal microscopy evaluation of Cy5-labeled aptamer sequence (6) binding to CEA-positive and CEA-negative cells. Red fluorescent signal was generated by Cy5-labeled aptamers. (**a**) Unstained CEA-positive HT-29 cell line; (**b**) HT-29 cell line treated with Cy5-labeled aptamer (6); (**c**) unstained CEA-negative HeLa S3 cells; (**d**) HeLa S3 cells treated with Cy5-labeled aptamer (6).

Confocal microscopy was used to determine whether aptamer (6) would recognize CEA expressed on HT-29 colorectal cancer cells. As shown in Figure 6b, HT-29 cells generated red fluorescent signals on the cell membrane, so we suppose that is directly the site of expression of the target protein CEA [50]. As a result, the red fluorescent signals from the Cy5-labeled novel aptamer (6) were strong in CEA-positive HT-29 cells (Figure 6b), and no signal was observed in the HeLa S3 cells that were used as a negative control (Figure 6d), further indicating that the novel aptamer (6) can bind to CEA-positive colorectal cancer cells in vitro.

## 4. Discussion

In this study, we performed in vitro selection with a pool of ssDNA sequences totally randomized at 40 nt to select DNA aptamers that bind to CEA. The ssDNA library had about 10^14^ different molecules with different sequences. As a result of twelve SELEX cycles, nine aptamer candidates were developed. The selected sequences were further described by bioinformatics analysis and ELONA. Multiple alignment of aptamer sequences enabled us to identify the common patterns between them, as well as to assess conservative regions in the structures and build a cladogram to present the putative relationship between aptamer sequences. During the screening of all nine aptamer sequences using ELONA, a sequence with the highest affinity and sensitivity towards CEA was identified, which was aptamer sequence (6). Being an alternative form of the conventional ELISA method, ELONA is a quick, cost-effective, and easy approach used to functionally characterize aptamers [34,53,54]. A fluorescent or colorimetric output was used when ELONA was developed many years ago and permitted simultaneous study of several aptamers [55,56]; however, for characterization of larger populations, fluorescently labeled DNA or RNA aptamers are a costly alternative. In this case, DNA aptamers may be easily adapted to colorimetric reading via biotin labeling [57], as in our study. Aptamer (6) was studied in different concentrations and analyzed for specificity, where IL-6 and HSA proteins were used as non-target molecules. IL-6 is promptly and transiently produced in response to infections and tissue injuries and contributes to host defense through stimulation of acute phase responses, hematopoiesis, and immune reactions [58]. HSA is a multifunctional, non-glycosylated, negatively charged extracellular plasma protein [59]. Albumin is the most abundant plasma protein in plasma, and its main function is maintaining the colloid osmotic pressure of plasma [60,61]. Bioinformatics analysis of aptamer (6) was also carried out; namely, possible secondary and tertiary structures were predicted. An aptamer’s secondary structure is essential in analyzing the binding capabilities to a target molecule and aids in prediction of the aptamer’s three-dimensional structure [62,63,64]. Molecular docking simulation showed us one of the theoretically possible interactions of aptamer sequence (6) with the protein complex. The sequence selectivity of aptamer (6) was proven using confocal microscopy imaging. To accomplish this, we tested the fluorescence upon binding of Cy5-tagged aptamer (6) to CEA on HT-29 cell surfaces [65]. According to Ref. [50], the HT-29 cell line expresses the CEA protein on its cell membrane, and this was confirmed in Figure 6b. The red fluorescence in the cell membrane showed selective binding of Cy5-labeled aptamer (6) to CEA, which is synthesized on the cell membrane. The selected aptamer sequence demonstrated specificity and sensitivity towards the target, which could potentially be used further in SPR- or electrochemical-impedance-spectroscopy (EIS)-based aptasensors development for CEA detection. Moreover, the current study contributes to the field of already available aptamers for CEA detection. For example, in Ref. [31], aptamers specific to the CEA protein were selected for their further use as radiopharmaceuticals for detection of colorectal cancer. Ref. [66] described development of two functional DNA aptamers chosen to bind the recombinant version of the IgV-like N domain of CEA and demonstrated its capability to prevent development of peritoneal tumor nodules from CEA-expressing tumor cells in vivo and disrupt CEA-mediated cellular interactions. Given the results obtained in our study, future work on aptamers modification for affinity and sensitivity, as well as specificity enhancement, could be completed by manner of sequence optimization, structure manipulation, and additional nucleotides incorporation [67]. Aptamers could be modified with magnetic/gold nanoparticles or fluorescent reporting molecules in case of weak signal. Sandwich assay with employment of two aptamer sequences or with an antibody could be conducted to enhance specificity and sensitivity of protein detection. For enhanced aptasensor applications, NMR, additional bioinformatics, and aptamer modification research could be carried out. This presents a significant opportunity for development of accurate, affordable, and rapid techniques for detection of CEA, where aptamers can be employed. 

## 5. Conclusions

In summary, nine ssDNA aptamer sequences against CEA were selected and characterized. The most sensitive and higher affinity binding towards the target protein was observed in aptamer sequence (6). This enabled us to study this aptamer sequence in a concentration–response manner and conduct a specificity study. The specificity of novel CEA aptamer (6) will be further enhanced using techniques such as sequence optimization and nucleotide incorporation. Molecular docking simulation analysis helped us to visualize and better understand the interaction between the aptamer sequence and CEA protein complex. The confocal microscopy results visually verified the binding of aptamer sequence (6) with the CEA target protein. The novel aptamer sequences selected in this study could potentially be used as biorecognition molecules in development of aptasensors used for detection of CEA. 

## Figures and Tables

**Table 1:** Amount of ssDNA and target protein used during SELEX procedure for in vitro selection of aptamers against CEA.

SELEX Cycle	Amount of Target Protein (CEA)		Amount of ssDNA		nM Ratio CEA/ssDNA	Incubation Time, min
	μg/mL	nM	ng/µL	nM		
1	45	250	1000	40466	1/0.2	105
2	45	250	22.1	894.3	1/4	90
3	7	38.9	32.8	1327	1/34	60
4	4.9	27.2	21.9	886.2	1/32	60
5	2	11.1	14	566.5	1/50	45
6	1.5	8.33	10	404.7	1/48	30
7	1.3	7.22	8	323.7	1/45	25
8	1.5	8.33	5	202.3	1/25	20
9	1.09	6.06	4.5	182.1	1/30	20
10	0.85	4.72	4	161.9	1/35	15
11	0.65	3.61	3.5	141.6	1/40	10
12	0.47	2.61	3	121.4	1/45	10

## Data Availability

Not applicable.

## Supplementary Materials

The following supporting information can be downloaded at: https://www.mdpi.com/article/10.3390/biology11101540/s1, Figure S1: Dot blot analysis of affinity binding between aptamer pools from the SELEX cycles of 12, 10, and 8 and target CEA, and two controls: “+” control was biotin (diluted at 1:500), “-” control—deionized water; Figure S2: 3% agarose gel of SELEX rounds 12 and 8 products corresponding to 90 bp; Table S1: The absorbance measurement of SELEX products.
