# Supplementary material for: A Novel ssDNA Aptamer Targeting Carcinoembryonic Antigen: Selection and Characterization
Source: Biology (Basel). 2022 Oct 20;11(10):1540. doi: 10.3390/biology11101540 (PMC9598387; doi:10.3390/biology11101540)
Supplement: Supplementary file 1 [file biology-11-01540-s001.zip › biology-1950278-supplementary.pdf]

Supplementary materials

Methods

Dot blot was used as a rapid analysis of affinity of the pool of aptamers from selected SELEX rounds and served as an alternative method for affinity confirmation. CEA and biotin were applied onto a nitrocellulose membrane (BA85 Protran, 0.45 µm, Whatman, USA) at 45 µg/ml concentration and air dried. The membrane surface was then blocked with Superblock solution for 60 min and washed 3 - 4 times with 1 × TBS buffer. The membrane was then air dried and biotinylated aptamer pools from the SELEX cycles of 12, 10, and 8 were left to incubate for 30 min. After washing three times with 1 × TBS the membrane reacted with streptavidin-alkaline phosphatase diluted to 1:500 for 30 min. The membrane was then coated in TMB substrate for 15 min in the dark after washing 3 - 4 times with 1 × TBS. The results were determined by observation of stained spots on the membrane. Biotin served as a positive control, and deionized water served as a negative control.

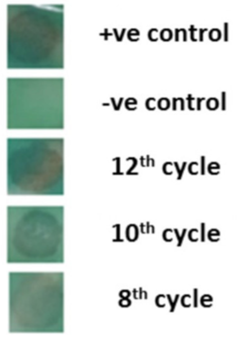

Figure S1. Dot blot analysis of affinity binding between aptamer pools from the SELEX cycles of 12, 10, and 8 and target CEA, and two controls: "+" control was biotin (diluted at 1:500), "-" control – deionized water.

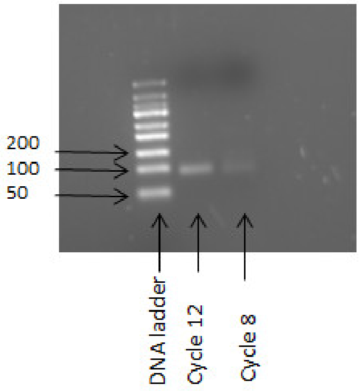

Figure S2. 3 % agarose gel of SELEX rounds 12 and 8 products corresponding to 90 bp.

Table S1. The absorbance measurement of SELEX products.

Round 8 DNA	A260 = 0.228	A260/280 = 1.9	11.4 ng/uL
Round 12 DNA	A260 = 0.260	A260/280 = 1.8	13 ng/uL
